# Needle, knife, or device – which choice in an airway crisis?

**DOI:** 10.1186/1757-7241-21-49

**Published:** 2013-06-27

**Authors:** Kate Crewdson, David J Lockey

**Affiliations:** 1 North Bristol NHS Trust, Bristol, UK; 2 London’s Air Ambulance, London, E1 1BB, UK

## 

Emergency cricothyroidotomy is a life saving intervention included as the final step in most guidelines addressing the management of the emergency airway [1,2]. The procedure must performed quickly and effectively to prevent death or hypoxic brain injury. Despite this a number of alternative approaches are recommended. Cricothyroidotomy can be performed using a needle or surgical approach, or with one of a large number of commercially available kits. Langvad and co-workers are to be congratulated on a comprehensive and systematic review of this controversial topic recently published in this journal [3]. The authors compared nine different combinations of cricothyroidotomy technique to summarise the available evidence. However, as is often the case in emergency medicine, systematic review of low-grade evidence does not necessarily equip the emergency physician with clear direction when faced with the emergency patient. The authors recognise that, given the considerable heterogeneity and poor quality of the studies, it was impossible to draw meaningful conclusions. No technique was demonstrated to be superior to the others. This conclusion does not mean that all techniques have similar success rates and complications, but that a limited amount of flawed evidence makes interpretation impossible.

The majority of studies are simply small pre-hospital or in-hospital case series describing the use of surgical airways. Most describe a surgical technique; only a handful of studies report the use of needle cricothyroidotomy. Kits are broadly divided into ‘puncture’, using a Seldinger method with a guide wire, or an ‘open’ technique, where a scalpel is used to incise the cricothyroid membrane. The study methodology is variable and generalisation is difficult. The procedures are performed by different healthcare providers, with different skill and training levels using a variety of kits and techniques on cadavers, animal models, or mannequins. The endpoints also range from time taken to insertion, or time taken to first effective ventilation, including or excluding failure or misplacement, to qualitative indices such as operator satisfaction or ease of use. There are few reports of kit use in real cases. New cricothyroidotomy devices are appearing regularly on the market, often with slightly different characteristics. The number and variation of available devices makes it difficult to provide adequate training for all types, and healthcare personnel (particularly those who rotate regularly through different hospitals) may need to use an unfamiliar one in an emergency situation, with the potential for poor performance.

In 2010, Hubble et al. published a meta-analysis of pre-hospital airway management, reporting success rates of 65.8% for needle cricothyroidotomy and 90.5% for surgical cricothyroidotomy [4]. The different methodology of this review means that these findings are not exactly reproduced by Langvad et al., but the majority of published work does suggest that surgical cricothyroidotomy is more likely to be successfully performed and more effective than a needle technique, with a minority of studies suggesting no difference between the techniques. There are also reports of successful surgical technique following failed needle or cannula cricothyroidotomy [5,6]. The catastrophic implications of needle cricothyroidotomy failure were brought into focus by an analysis of closed insurance claims for difficult airway management in the US [6]. One hundred and seventy-nine claims were analysed, of which 75 required perioperative emergency rescue airway management. Surgical airway was attempted in 57 patients (76%), but of these, 48 patients (84%) suffered death or significant brain damage. In many cases the procedure was successful but performed too late to avoid a poor outcome. Needle cricothyroidotomy was attempted in 26 patients, all of which had a poor outcome. In 89% of cases where jet ventilation was used, the patient developed pneumothorax, pneumomediastinum, or subcutaneous emphysema. Surgical cricothyroidotomy was often carried out after failed needle cricothyroidotomy and was more successful. Although the analysis of closed claims inevitably only focuses on those patients who come to harm this ‘real patient’ data is of major concern.

Other recent publications have also questioned the role of needle cricothyroidotomy [7-9]. The fourth UK National Audit Project of the Royal College of Anaesthetists and Difficult Airway Society (NAP 4), recorded major complications of airway management. It reported a small number of cases where needle cricothyroidotomy performed by anaesthetists had a very low success rate (37%) [7]. Current European Resuscitation Council guidelines suggest needle cricothyroidotomy should only be considered a temporary measure, in contrast to surgical cricothyroidotomy which provides a definitive airway [8].

Advances in airway training and equipment and the use of algorithms, has been reported to reduce failed intubation rates [10]. Emergency cricothyroidotomy is therefore performed very infrequently. Despite this, personnel who will usually be performing the procedure for the first time have to be able to do so rapidly and effectively. It is possible that surgical cricothyroidotomy is performed by those who are most likely to succeed with this technique, perhaps because of prior training. In NAP4 surgical airway was often successful, but was frequently performed by surgeons [7]. Although anaesthetists are familiar with needle techniques and might be expected to excel at needle cricothyroidotomy, the published literature does not support this theory. It is difficult to judge the effectiveness and relevance of the different training models to actual clinical practice, but it does seem that training is important in achieving competence and speed in the basic techniques. Time is critical; training people to act confidently and quickly is essential. The time taken to make the decision to perform an emergency cricothyroidotomy is likely to be more important than the technique used.

Where does that leave us now? Whilst this excellent review provides a comprehensive picture of the evidence base for emergency cricothyroidotomy, the low quality of the evidence makes it difficult to provide clear guidance for the practitioner who finds themselves in a can’t intubate, can’t ventilate situation. The nature of this type of emergency does not allow indecision. On the basis of the available evidence we have adopted the following position in our pre-hospital service. There is no good evidence supporting the use of needle cricothyroidotomy, but plenty of low quality evidence that it is often associated with failure and complications, and may delay oxygenation. There is also currently no good evidence that any commercially produced kit is better than a standard surgical technique. Standard surgical airway is a reliable technique with good success rates.

The speed of re-oxygenation is of critical importance to outcome. Delay produced by performing a needle cricothyroidotomy then having to convert to a surgical airway may be the difference between a good outcome and severe disability or death. A failed needle technique may also make a subsequent successful surgical cricothyroidotomy harder to achieve, particularly where surgical emphysema or barotrauma has occurred. We believe that training should concentrate on early recognition of the situation in which surgical airway is required and then rapid performance of a standard surgical technique that does not rely on the availability of potentially unfamiliar equipment. This approach is likely to reduce procedural error or failure seen with kits, cannulae, or jet ventilation and is a valid and pragmatic approach to the important lessons reported in key relevant publications [6,7].

The authors believe that future emergency airway management algorithms should strongly consider promotion of a primary surgical airway technique over needle techniques and commercially produced emergency airway kits.
